# Cardiomiopatia Chagásica Na Amazônia Brasileira: Baixa Prevalência Ou Subdiagnóstico?

**DOI:** 10.36660/abc.20201236

**Published:** 2021-10-06

**Authors:** Jessica Vanina Ortiz, Katia do Nascimento Couceiro, Susan Smith Doria, Débora Raysa Teixeira de Sousa, Henrique Manuel Condinho da Silveira, Norival Kesper, Maria das Graças Vale Barbosa Guerra, Jorge Augusto de Oliveira Guerra, João Marcos Bemfica Barbosa-Ferreira

**Affiliations:** 1 Programa de Pós-Graduação em Medicina Tropical Escola de Ciências da Saúde Universidade do Estado do Amazonas ManausAM Brasil Programa de Pós-Graduação em Medicina Tropical, Escola de Ciências da Saúde, Universidade do Estado do Amazonas, Manaus, AM - Brasil; 2 Instituto de Higiene e Medicina Tropical Lisboa Portugal Instituto de Higiene e Medicina Tropical, Lisboa - Portugal; 3 Hospital das Clínicas FMUSP São PauloSP Brasil Hospital das Clínicas da FMUSP-LIM49, São Paulo, SP - Brasil; 4 Fundação de Medicina Tropical Dr. Heitor Vieira Dourado ManausAM Brasil Fundação de Medicina Tropical Dr. Heitor Vieira Dourado, Manaus, AM - Brasil; 5 Fundação Hospital do Coração Francisca Mendes ManausAM Brasil Fundação Hospital do Coração Francisca Mendes, Manaus, AM - Brasil

**Keywords:** Doença de Chagas, Insuficiência Cardíaca, Cardiomiopatia Chagásica

## Introdução

A doença de Chagas (DC) foi descoberta em 1909 pelo médico brasileiro Carlos Chagas, que descreveu o agente etiológico – um protozoário flagelado denominado *Trypanossoma cruzi* – suas características morfológicas e seu ciclo de vida e de transmissão, bem como manifestações clínicas da doença.^1^ Apesar de os insetos triatomíneos serem os vetores primários de transmissão da DC, a via oral por meio de alimentos contaminados foi considerada o meio principal de transmissão na Amazônia brasileira.

A doença apresenta duas fases clínicas: uma infecção aguda, predominantemente assintomática com elevada parasitemia, e uma infecção crônica dividida em forma indeterminada assintomática ou forma sintomática (digestiva ou cardíaca).^2^

A cardiomiopatia chagásica crônica (CCC) é caracterizada por uma cardiomiopatia dilatada causada por uma inflamação crônica, e é a principal causa de morte entre pacientes com cardiomiopatia não isquêmica na América Latina.^3 , 4^ A Organização Mundial da Saúde estima que haja oito milhões de pessoas infectadas em todo o mundo, e aproximadamente 232 mil pessoas com CCC no Brasil.^2^ Há poucos estudos publicados com indivíduos com DC crônica e CCC na região da Amazônia brasileira. O primeiro relato de casos crônicos foi realizado em 1977,^5^ e o do primeiro caso de CCC em 2003.^6^ Xavier et al.^7^ e Ferreira et al.^8^ também relataram novos casos de DC crônica e CCC. Em dez anos, nenhum estudo atualizou as características epidemiológicas de CCC na região.

O diagnóstico de DC crônica é feito por dois testes sorológicos diferentes: ensaios imunoenzimáticos (ELISA), imunofluorescência indireta (IFI). Exame parasitológico, teste de biologia molecular, e *western blot* são usados como métodos complementares. Técnicas de biologia molecular têm sido amplamente utilizadas para identificar a linhagem do *T. cruzi* circulante na região.^2 , 9^ O presente estudo teve como objetivo avaliar a prevalência de DC em pacientes com cardiomiopatia dilatada de causa desconhecida na região da Amazônia brasileira para tentar responder as seguintes perguntas: a CCC está sendo subdiagnosticada? Ou, existe uma baixa prevalência da doença em nossa região?

## Métodos

### Locais do estudo

Este estudo foi um estudo transversal conduzido no Hospital Universitário Francisca Mendes (Unidade de Cardiologia) e na Fundação de Medicina Tropical Doutor Heitor Vieira Dourado (doenças infecciosas / unidade de doença de Chagas), dois centros de atenção terciária especializados no estado do Amazonas, Brasil.

### População do estudo

Os participantes foram recrutados de julho 2017 a julho 2018 e foram considerados elegíveis se apresentassem: 1) idade ≥ 18 anos; 2) perfil cardíaco de cardiomiopatia dilatada idiopática, em que qualquer outra causa de cardiomiopatia já tiver sido excluída; 3) resultado anormal no ecocardiograma (fração de ejeção do ventrículo esquerdo [FEVE] reduzido e/ou alterações no segmento) e/ou no eletrocardiograma (bloqueio de ramo, bloqueio atrioventricular, ou arritmias).

Os seguintes fatores de risco epidemiológicos foram considerados: 1) ser originário da região da Amazônia brasileira; 2) ser originário de áreas rurais; 3) ter hábito de ir à floresta; e 4) consumir frutos de palmeiras e/ou carne de animais silvestres.

Pacientes com qualquer evidência de cardiomiopatia isquêmica ou congênita, ou de doença valvular cardíaca não foram elegíveis para inclusão. Ainda, os participantes que relataram ter viajado a outra região brasileira ou a um país estrangeiro foram excluídos do estudo.

### Coleta de dados e de amostras de sangue

Todos os pacientes que preencheram os critérios de inclusão foram convidados a participar e, aqueles que aceitaram, assinaram o termo de consentimento. Os participantes foram primeiramente avaliados usando um questionário clínico e epidemiológico. Em seguida, as amostras de sangue foram coletadas, centrifugadas, separadas e armazenadas a -20^o^C.

### Exames sorológicos

O diagnóstico de DC foi realizado por ELISA (Chagatest ELISA recombinante v. 4.0, Wiener Laboratorios, Argentina) e imunofluorescência indireta (Imuno-CON Chagas, WAMA Diagnostica, Brasil), seguindo-se as instruções dos fabricantes.

Para resultados indeterminados, utilizou-se o teste de *immunoblotting* para análise de antígenos secretados e excretados por tripomastigota (TESA, do inglês *trypomastigote excreted-secreted antigen* ), o TESA-blot. As frações de TESA foram obtidas a partir do sobrenadante de células MK2 infectadas pela cepa Y de *T. cruzi* , seguindo o protocolo descrito previamente.^10^

### Detecção e caracterização molecular do T. cruzi

A extração de DNA foi realizada a partir de sangue periférico, seguindo-se o protocolo do kit comercial PureLink® Genomic DNA Mini (Invitrogen, Life Technologies, California, EUA). Este método foi realizado como um teste complementar para soros com resultados indeterminados nos exames sorológicos, para aumentar a acurácia diagnóstica.^11^

As amostras foram submetidas à tipagem do DNA mitocondrial pela análise de polimorfismos nos genes que codificam a subunidade II(COII).^12^ Os produtos de amplificação de PCR foram purificados utilizando-se o sistema Wizard SV Gel and PCR Clean-up System kit (Promega) e o sequenciamento realizado pelo sequenciador de DNA ABI 3130 (Applied Biosystems). Seguimos o protocolo do kit de sequenciamento BigDye Terminator v3.1 (Applied Biosystems) e sequências das cepas padrões: TcI (Silvio X10 cl1), TcII (Esmeraldo cl3), TcIII (M6241 cl6), TcIV (CANIII cl1), TcV (Mn cl2), e TcVI (CL Brener). A análise de evolução foi conduzida no MEGA X.^13^

### Aspectos éticos

O estudo foi aprovado pelo comitê de ética em pesquisa da Fundação de Medicina Tropical Doutor Heitor Vieira Dourado (Manaus, AM, Brasil) (número 69904017.9.0000.0005-2.191.571/28, julho 2017), em concordância com a resolução 466/12 do Conselho Nacional de Saúde e diretrizes éticas da Declaração de Helsinki de 1975.

### Apresentação e análise dos dados

Os dados clínicos e epidemiológicos foram organizados utilizando o Microsoft Excel 2016 e a análise descritiva realizada utilizando o Stata/MP 13.0. As variáveis categóricas foram descritas em frequências e proporções (%), e as variáveis contínuas em médias e desvios padrões (DP).

## Resultados

### Prevalência da doença de Chagas

Foram incluídos 53 pacientes, e oito foram excluídos – um morreu antes da coleta de sangue, dois comentaram ter morado em outro local, e cinco não compareceram à coleta de sangue.

Nas 45 amostras viáveis, foram realizados os dois testes sorológicos, o ELISA e a imunofluorescência indireta. Uma amostra foi reagente no teste ELISA mas não reagente na imunofluorescência indireta, e 13 foram reativos na imunofluorescência indireta. Essas 14 amostras (31%) foram submetidas ao teste TESA-blot, e todos deram negativos. Assim, nenhum dos casos suspeitos de CCC foi confirmado por método sorológico.

### Detecção e caracterização molecular do T. cruzi

**Figura 1:**
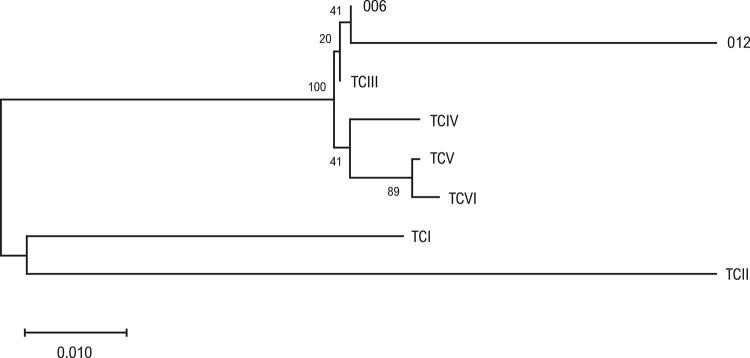
– *Posição filogenética do Trypanosoma cruzi nas amostras 006 e 012 baseada nas sequências genéticas da subunidade II da citocromo oxidase.*

As amostras que apresentaram pelo menos uma reação sorológica positiva foram submetidas à análise molecular; dois foram positivas e identificadas como TcIII/IV ( Figura 1 ).

Dados clínicos e laboratoriais desses dois pacientes estão resumidos na Tabela 1 .

**Tabela 1:** – Características clínicas e demográficas de todos os casos suspeitos de cardiomiopatia chagásica

Variável	Total (n=45)
Idade (anos)	59,3 ± 12,3
**Sexo**	
Masculino	35 (77,8%)
Feminino	10 (22,2%)
**Área**	
Rural	34 (75,6%)
Urbana	11 (24,4%)
**Origem**	
Acre	4 (9,0%)
Amazonas	32 (71,1%)
Maranhão	1 (2,2%)
Pará	6 (13,3%)
Roraima	2 (4,4%)
**Fatores de susceptibilidade**	
Agricultura/extrativismo/pescaria	27 (60,0%)
Consumo de carne de caça	32 (71,1%)
Consumo de fruto do açaizeiro	30 (66,7%)
Hábito de entrar na floresta	30 (66,7%)
**Eletrocardiograma**	
Bloqueio de ramo direito	2 (4,4%)
Bloqueio de ramo esquerdo	4 (8,9%)
Bloqueio fascicular anterior esquerdo	2 (4,4%)
Fibrilação atrial	3 (6,7%)
Repolarização ventricular	21 (46,7%)
Normal	13 (28,9%)
**Ecocardiograma transtorácico**	
FEVE (%)	30,3 ± 10,7
Diâmetro diastólico do ventrículo esquerdo (mm)	64,6 ± 9,1
Acinesia localizada	7 (15,6%)
Parede anterior septal	3 (42,8%)
Parede lateral inferior	2 (28,6%)
Parede septal inferior	1 (14,3%)
Parede inferior	1 (14,3%)
Hipocinesia difusa	11 (24,4%)
**Comorbidades**	
Diabetes mellitus	9 (20,0%)
Hipertensão	22 (49,0%)

*Dados expressos em média ± desvio padrão e porcentagens; FEVE: fração de ejeção do ventrículo esquerdo.*

## Discussão

### Prevalência da doença de Chagas

Por muito tempo, acreditou-se que não existiam casos de DC na Amazônia brasileira. No entanto, isso mudou ao longo dos anos com relatos de muitos casos de DC aguda e crônica.

De acordo com o consenso brasileiro, o diagnóstico de DC deve ser confirmado pela combinação de dois métodos sorológicos. Estudos prévios realizados no estado do Amazonas^14^ relataram uma baixa prevalência de baixa morbidade da DC, e discutiram a provável baixa eficácia dos testes sorológicos comerciais disponíveis para as cepas circulantes, considerando que esses testes são realizados com diferentes cepas.^11^

Até o momento, foram relatados oito casos de CCC,^6 - 8^ e quatro desses pacientes (50%) apresentaram aneurisma apical, uma frequência esperada de acordo com estudos com pacientes de áreas endêmicas.^15^ Ferreira et al.^8^ estudaram pacientes com disfunção sistólica do ventrículo esquerdo de causa desconhecida, e encontraram uma prevalência de CCC de 8,1%.

Em nosso estudo, os dois pacientes que apresentaram resultado de DNA positivo não apresentaram aneurisma, e um deles apresentou achados eletrocardiográficos normais. Um ECG normal não é comum (5%) segundo estudos prévios, mas é possível mesmo na presença de uma doença cardíaca clínica ou disfunção no ecocardiograma.^16 , 17^ Vale destacar, no entanto, que a presença de DNA do *T. cruzi* pode não ser suficiente para confirmar CCC, e não podemos excluir uma possível coincidência na identificação do genótipo do parasita no paciente com disfunção ventricular grave, sem aneurisma apical ou diagnóstico sorológico, e com um ECG normal. O segundo paciente com DNA positivo apresentou bloqueio de ramo direito e bloqueio fascicular anterior esquerdo no ECG. Nesse paciente, a presença de DNA de *T. cruzi* e alterações típicas no ECG torna o diagnóstico de CCC possível mesmo sem diagnóstico sorológico.

Embora o presente estudo não tenha identificado novos casos de CCC, como proposto inicialmente, nossos resultados levantam algumas questões: a doença de Chagas crônica, e consequentemente a CCC, é ainda subdiagnosticada na região da Amazônia brasileira? Os métodos sorológicos disponíveis têm se tornado menos eficiente em relação às cepas circulantes do parasita nesta região?

### Detecção e caracterização molecular do T. cruzi

Apesar de técnicas moleculares não terem sido utilizadas no diagnóstico de DC devido a possíveis resultados falso positivos, o método foi incluído no estudo com base em estudos prévios que corroboram sua especificidade em infecções crônicas, e seu papel como um método complementar em casos em que resultados nos testes sorológicos persistirem inconclusivos.^18^ Nós identificamos o DNA do parasita em duas amostras, e determinamos seu genótipo como TcIII/TcIV.

Esta é a primeira vez em que a cepa TcIII/TcIV do *T. cruzi* é encontrada em uma fase crônica da doença. Este fato pode estar relacionado com a presença de TcIII nos vetores e reservatórios, e TcIV na fase aguda da DC, conforme relatado previamente na região.^11 , 19^ Outros estudos mostraram uma reatividade satisfatória ao teste sorológico e TESA-blot em regiões endêmicas no Brasil e no Panamá.^9^ No entanto, devido à região amazônica ainda ter uma epidemiologia particular, todos os recursos disponíveis foram usados neste estudo a fim de chegar a um resultado satisfatório para o paciente, incluindo uma PCR qualitativa reativa.^20^

### Limitações do estudo

O pequeno tamanho amostral limitou nossa capacidade de demonstrar o real cenário da CCC em nossa região. Similarmente, temos visto uma baixa reatividade dos kits sorológicos comerciais disponíveis e uma reatividade cruzada moderada com outras doenças endêmicas.

## Conclusões

Uma baixa prevalência de CCC foi observada neste grupo de participantes. Em duas das 45 amostras (4%), fragmentos de DNA de *T. cruzi* foram detectados seguindo-se protocolos rígidos. No entanto, é importante lembrar que a maioria das amostras de soro de pacientes desta região tem baixa reatividade sorológica e moderada reatividade cruzada com outras doenças, o que torna difícil o diagnóstico. Assim, é muito importante a continuidade de pesquisas clínicas e epidemiológicas para determinar a real situação da CCC na região da Amazônia.
