# Supplementary material for: Cardiomiopatia Chagásica Na Amazônia Brasileira: Baixa Prevalência Ou Subdiagnóstico?
Source: Arq Bras Cardiol. 2021 Oct 6;117(4):770–4. [Article in Portuguese] doi: 10.36660/abc.20201236 (PMC8528347; doi:10.36660/abc.20201236)
Supplement: Translation

# Translation: Chagas Cardiomyopathy in the Brazilian Amazon Region: Low Prevalence or Underdiagnosis?

**Authors:** Jessica Vanina Ortiz, Katia do Nascimento Couceiro, Susan Smith Doria, Débora Raysa Teixeira de Sousa, Henrique Manuel Condinho da Silveira, Norival Kesper, Maria das Graças Vale Barbosa Guerra, Jorge Augusto de Oliveira Guerra, João Marcos Bemfica Barbosa-Ferreira

**Affiliations:** 1 Brasil Programa de Pós-Graduação em Medicina Tropical, Escola de Ciências da Saúde, Universidade do Estado do Amazonas, Manaus, AM - Brasil; 2 Portugal Instituto de Higiene e Medicina Tropical, Lisboa - Portugal; 3 Brasil Hospital das Clínicas da FMUSP-LIM49, São Paulo, SP - Brasil; 4 Brasil Fundação de Medicina Tropical Dr. Heitor Vieira Dourado, Manaus, AM - Brasil; 5 Brasil Fundação Hospital do Coração Francisca Mendes, Manaus, AM - Brasil

**Keywords:** Chagas Disease, Heart Failure, Chagas cardiomyopathy. Chagas Cardiomyopathy

## Introduction

Chagas disease (CD) was discovered in 1909 by the Brazilian physician Carlos Chagas, who described the etiological agent – a flagellated protozoan named *Trypanosoma cruzi* ( *T. cruzi* ) – its morphological features and transmission and life cycle, as well as clinical manifestations of the disease.^1^ Although triatomine insects are the primary transmission vectors of CD, the oral route through contaminated food has been considered the main transmission path in the Brazilian Amazon.

The disease presents two clinical phases: an acute infection, mostly symptomatic with high parasitemia, and a chronic infection divided into asymptomatic indeterminate form or symptomatic (digestive or cardiac) form.^2^

Chronic Chagas cardiomyopathy (CCM) is characterized by a dilated cardiomyopathy due to the long-term inflammation and is the main cause of death of patients with nonischemic cardiomyopathy in Latin America.^3 , 4^ The World Health Organization estimates eight million infections worldwide, and about 232 thousand people with CCM in Brazil.^2^ In the Brazilian Amazon region, little has been published about chronic CD and CCM; the first report of chronic cases was made in 1977,^5^ and the first case of CCM reported in 2003.^6^ Xavier et al.^7^ and Ferreira et al.^8^ have also reported new cases of chronic CD and CCM. In ten years, no new report has updated the epidemiological features of CCM in the region.

The diagnosis of chronic CD is made by two different serological tests: enzyme-linked immunosorbent assay (ELISA), indirect immunofluorescence (IIF). Parasitological test, molecular biology test, and western blotting are used as complementary methods. Molecular biology techniques have been widely used to genotype the *T. cruzi* strain circulating in the area.^2 , 9^

Therefore, this study aimed to evaluate the prevalence of CD in patients with dilated cardiomyopathy of unknown etiology in the Brazilian Amazon region to try to answer the questions: is CCM being underdiagnosed? Or rather, is there a low prevalence of this disease in our region?

## Methods

### Study sites

This was a cross-sectional study conducted at Francisca Mendes University Hospital (Cardiology Unit) and Dr. Heitor Vieira Dourado Tropical Medicine Foundation (Infectious Diseases/Chagas Disease Unit), two specialized tertiary care centers in the state of Amazonas, Brazil.

### Study Population

Participants were recruited from July, 2017 to July, 2018 and were eligible for inclusion if: 1) were aged ≥ 18 years, 2) had a cardiac profile of idiopathic dilated cardiomyopathy, and any other cause of cardiomyopathy had been already excluded, 3) had abnormal echocardiogram (reduced left-ventricular ejection fraction [LVEF] and/or segmental alterations), and/or electrocardiogram (bundle branch block, atrial ventricular block, or arrhythmias).

The following epidemiological risk factors were also considered: 1) being from the Brazilian Amazon; 2) coming from rural areas; 3) having the habit of entering the forest; and 4) consuming palm tree fruits and/or meat of wild mammals.

Patients with any evidence of ischemic or congenital cardiomyopathy or valve disease were not eligible for inclusion. Also, participants that reported a previous trip to another Brazilian region or foreign country were excluded from the study.

### Data and blood sample collection

All patients who met the inclusion criteria were invited to participate in the study, and those who agreed to participate signed the informed consent form. Participants were first evaluated using a clinical and an epidemiological questionnaire. Then, blood samples were collected, centrifuged, separated, and stored at -20ºC.

### Serological tests

The diagnosis of CD was performed by ELISA (Chagatest ELISA recombinante v. 4.0, Wiener Laboratorios, Argentina) and IIF (Imuno-CON Chagas, WAMA Diagnostica, Brazil), following the manufacturers’ instructions.

For indeterminate results, the TESA (trypomastigote excreted-secreted antigen)-blot test was performed. The TESA fractions were obtained from the supernatant of MK2 cells infected with T. cruzi Y strain, following the protocol as previously described.^10^

### T. cruzi molecular detection and characterization

DNA extraction was done using peripherical blood and following the protocol of the commercial kit PureLink™ Genomic DNA Mini (Invitrogen, Life Technologies, California, USA). This method was performed a complementary test for sera with indeterminate serological test results, in order to increase diagnostic accuracy.^11^

Samples were subjected to mitochondrial DNA typing by analysis of polymorphisms in the cytochrome oxidase subunit II (COII) genes.^12^ The amplified PCR products were purified by using the Wizard SV Gel and PCR Clean-up System kit (Promega) and sequencing performed with ABI 3130 DNA sequencer (Applied Biosystems).

We followed the BigDye Terminator v3.1 Cycle Sequencing Kit protocol (Applied Biosystems) and sequences from standard strains: TcI (Silvio X10 cl1), TcII (Esmeraldo cl3), TcIII (M6241 cl6), TcIV (CANIII cl1), TcV (Mn cl2), and TcVI (CL Brener). Evolutionary analysis was conducted in MEGA X.^13^

### Ethical Considerations

This study was approved by the Research Ethics Committee of Tropical Medicine Foundation Dr. Heitor Vieira Dourado (Manaus, AM, Brazil) (approval number 69904017.9.0000.0005-2.191.571/28, July 2017), in agreement with the Resolution 466/12 of the Brazilian National Health Council and ethical guidelines of the 1975 Declaration of Helsinki.

### Data presentation and analysis

Clinical and epidemiological data were organized using Microsoft Excel 2016 and a descriptive analysis was done using Stata/MP 13.0. Categorical variables were described as frequencies and proportions (%), and continuous variables as means and standard deviations (SD).

## Results

### Prevalence of Chagas disease

Fifty-three patients were included, and eight of them were excluded: one died before blood collection, two mentioned having lived elsewhere, and five did not show up for blood sample collection. Demographic and clinical characteristics of the 45 patients are described in Table 1 .

**Table 1:** – Demographic and clinical characteristics of all suspected cases of Chagas cardiomyopathy

Variable	Total (n=45)
Age (y)	59.3 ± 12.3
**Gender**	
Male	35 (77,8%)
Female	10 (22.2%)
**Area**	
Rural	34 (75.6%)
Urban	11 (24.4%)
**Origin**	
Acre	4 (9.0%)
Amazonas	32 (71.1%)
Maranhão	1 (2.2%)
Pará	6 (13.3%)
Roraima	2 (4.4%)
**Susceptibility factors**	
Agriculture/extractivism/fishing	27 (60.0%)
Game meat consumption	32 (71.1%)
Açai fruit consumption	30 (66.7%)
Habit to enter the forest	30 (66.7%)
**Electrocardiogram**	
Right bundle branch block	2 (4.4%)
Left bundle branch block	4 (8.9%)
Left anterior fascicular block	2 (4.4%)
Atrial fibrillation	3 (6.7%)
Ventricular repolarization	21 (46.7%)
Normal	13 (28.9%)
**Transthoracic echocardiogram**	
LVEF (%)	30.3 ± 10.7
Left ventricular diastolic diameter (mm)	64.6 ± 9.1
Localized akinesia	7 (15.6%)
Anterior septal wall	3 (42.8%)
Inferior lateral wall	2 (28.6%)
Inferior septal wall	1 (14.3%)
Inferior wall	1 (14.3%)
Diffuse hypokinesia	11 (24.4%)
**Comorbidities**	
Diabetes mellitus	9 (20.0%)
Hypertension	22 (49.0%)

*Data expressed as mean ± SD and percentages; LVEF: left ventricular ejection fraction.*

In 45 suitable samples, two serological tests were performed, ELISA and IIF. One was reactive in the ELISA but not reactive in the IIF, and 13 were reactive in IIF. In these 14 (31%) samples, TESA-blot confirmatory test was performed, and all came up with negative results. Thus, none of the suspected CCM was confirmed by serological methods.

### T. cruzi molecular detection and characterization

The samples that had at least one positive serological reaction were subjected to molecular analysis; two were positive and genotyped as TcIII/IV ( Figure 1 ). Clinical and laboratory data of these two patients are summarized in Table 2.

**Figure 1:** – *Phylogenetic position of Trypanosoma cruzi in samples 006 and 012 based on the cytochrome oxidase subunit II gene sequences.*

## Discussion

### Prevalence of Chagas disease

For a long time, it was believed that CD did not exist in the Brazilian Amazon. However, this has changed over the years as many cases of acute and chronic CD have been reported.

According to the Brazilian Consensus, the diagnosis of CD should be confirmed by the combination of two serological methods. Previous studies in the state of Amazonas^14^ have reported a low prevalence and low morbidity of Chagas disease, and discussed the probable low efficacy of commercially available serological tests for the strain circulating, considering that these tests are made with different strains.^11^

Eight cases of CCM have been reported until now,^6 - 8^ and four of these patients (50%) showed apical aneurysms, an expected frequency according to studies with patients from endemic areas.^15^ Ferreira et al.^8^ studied patients with left ventricular systolic dysfunction of unknown etiology, and found a prevalence of CCM of 8.1%.

In our study, the two DNA-positive patients had no apical aneurysm and one of these patients had normal ECG findings. A normal ECG is uncommon (5%) according to previous studies on Chagas heart disease, but it is possible even in the presence of a clinical heart disease or echocardiogram dysfunction.^16 , 17^ It is worth pointing out, however, that the presence of *T. cruzi* DNA may not be sufficient to confirm CCM, and we cannot exclude a possible coincidence in the identification of the parasite genotype in the patient with severe ventricular dysfunction, without apical aneurysm or serological diagnosis, and with a normal ECG. The second patient with positive DNA had right bundle branch block and left anterior fascicular block (typical findings of CCM) on ECG. In this patient the presence of *T. cruzi* DNA and typical ECG changes makes the diagnosis of CCM very possible even without of serological diagnosis.

Even though our study did not identify new cases of CCM, as initially proposed, these results raise some questions: Is chronic Chagas disease, and consequently, CCM still underdiagnosed in the Brazilian Amazon region? Have the serological methods available been losing efficiency in relation to the circulating strains of the parasite in this region?

### *T. cruzi* molecular detection and characterization

Despite the fact that molecular techniques have not been used for diagnosis of CD due to possible false-positive results, the method was included in this study based on previous studies supporting its specificity in chronic infections and its role as a complementary method where inconclusive serological techniques persist.^18^ We identified the parasite’s DNA in two samples and genotyped as TcIII/TcIV.

This is the first time that TcIII/TcIV strain of *T. cruzi* is found in a chronic phase of the disease. This fact may be related to the presence of TcIII in the vectors and reservoirs, and TcIV in the acute phase of CD, as previously reported in the region.^11 , 19^ Other studies have shown a satisfactory reactivity to serological and TESA-blot test in endemic regions in Brazil and Panamá.^9^ Nevertheless, due to the Amazon region still having a particular epidemiology, all available resourcer were used in this study in order to reach a satisfactory result for the patient, including a qualitative reactive PCR.^20^

### Study limitations

The small sample size in this work limited our ability to demonstrate the real scenario of CMM in our region. Similarly, we have seen a low reactivity of commercially available serological kits and a moderate cross-reactivity with other endemic diseases.

## Conclusions

A low prevalence of CCM was observed in this group of study participants. In two of 45 samples (4%) *T. cruzi* DNA fragments were detected following strict protocols. However, it is important to remember that most sera from patients from this location have low serological reactivity and moderate cross-reactivity with other diseases, which makes diagnosis difficult. Therefore, it is very important to continue epidemiological and clinical research to determine the real situation of CCM in the Amazon region.
